# Mucocele Accompanied by a Traumatic Neuroma: A Case Report

**Published:** 2013-03

**Authors:** Z Jaafari Ashkavandi, A Dehghani Nazhvani, M Hamzavi

**Affiliations:** a Dept. of Oral and Maxillofacial Pathology, School of Dentistry, Shiraz University of Medical Sciences, Shiraz, Iran; b Specialist in Oral and Maxillofacial Pathology, School of Dentistry, Shiraz University of Medical Sciences, Shiraz, Iran

**Keywords:** Labial Mucosa, Mucocele, Traumatic Neuroma

## Abstract

Mucocele and traumatic neuroma are two lesions related to the traumatic events; however there is only one reported case in which these two entities were perceived simultaneously. The current study reported a 21-year-old man who complained of painless recurrent swelling, accompanied by paresthesia on his left lower labial mucosa. He had a previous history of similar lesion and had been treated with surgery and cauterization last year. The primary clinical impression was a recurrent mucocele. Microscopic surveys displayed a traumatic neuroma in the vicinity of a mucocele which seems to be arising from the previous surgical treatment.

## Introduction

Mucocele is a common lesion in the oral cavity which develops after traumatic rupturing of a salivary gland duct and consequent accumulation of mucin in the surrounding soft connective tissue. The lesion is commonly described as a painless bluish, gray or normal-in-color swelling which may show intermittent rupture. The treatment of choice is surgery and if the feeding duct remains in place, the possibility of recurrence is high [1-2]. Surgical procedures or traumatic injuries may result in cutting a peripheral nerve fiber followed by an abnormal healing which leads to the formation of a traumatic neuroma [3]. Traumatic neuroma of the oral mucosa is mainly found in the gingiva after tooth extraction and it usually occurs in the elderly adults. Involvement of the lower lip is rare and only a few cases were reported in the literature [4]. As mentioned earlier, the major etiologic factor of these two lesions is trauma. Up to the present time, only one case of mucocele joined with traumatic neuroma was reported [5]. We represent the second case in which these two entities occurred simultaneously.

## Case report

**Figure 1:**
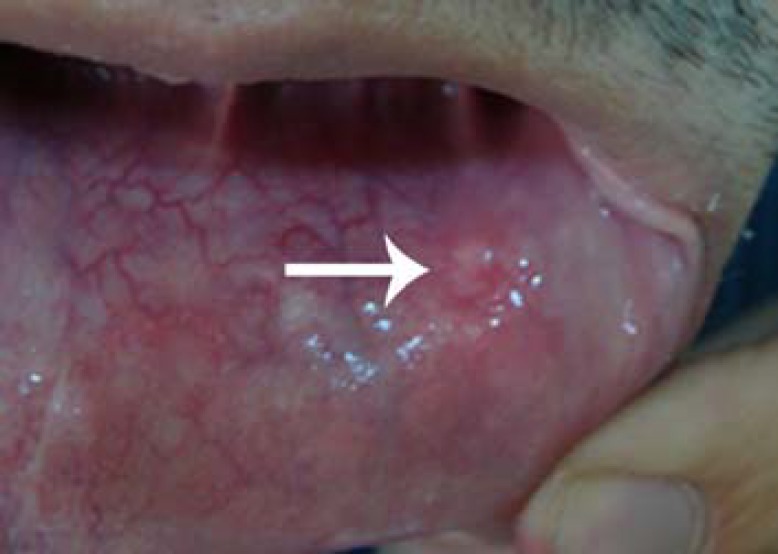
Hemispherical transparent swelling on the labial mucosa (arrow

A 21-year-old man presented who complained of a painless swelling with paresthesia on his left lower lip. He had a past history of similar lesion diagnosed as mucocele one year earlier, and the lesion was treated by surgery and electrocauterization. Eight months after his first surgery, a new swelling in the earlier place was aroused and remained for 4 months. The size of the lesion did not change during this period on the basis of what our patient reported. There was a hemispherical transparent swelling sized 1x0.7x0.3cm. It was firm and could not be easily ruptured by usual traumatic manipulations (Figure 1). 

**Figure 2a:**
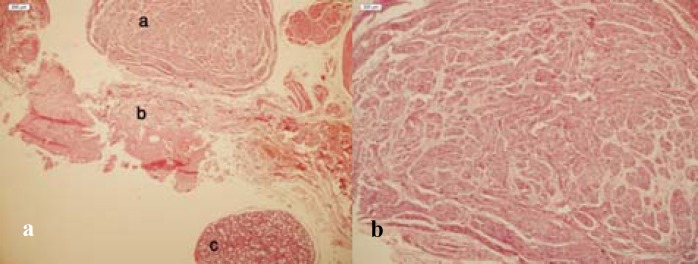
a Traumatic neuroma: haphazard arrangement of nerve bundles within the stroma b Mucocele: area of spilled mucin surrounded by granulation tissue c Minor salivary glands **b** Traumatic neuroma: nerve bundles within dense fibrous connective tissue (×100)

The clinical impression was consistent with recurrent mucocele, however paresthesia was not justified. The lesion was removed by excisional surgery. The surgical specimen consisted of multiple pieces of a cyst-like creamy elastic soft tissue. It comprised of some small round structures similar to the minor salivaryglands with one bigger than the others, totally measuring: 1x 0.8 x 0.7 cm. Microscopic examination revealed a pool of mucin surrounded by granulation tissues. Adjacent minor salivary gland showed mild sialoadenitis (Figures 2). Close to this area, a large irregular proliferation of the nerve fibers in a fibrous stroma was observed. According to these clinical and histopathological data, the diagnosis of mucocele accompanied with traumatic neuroma was established. Four months after the surgery, the patient was still suffering from paresthesia and vague pain, although the swelling subsided completely.

## Discussion

Oral mucosa has a rich nerve supply which lies in the underlying connective tissue or mucosal appendices such as minor salivary glands. Any surgical procedure is always traumatic to the hard and soft tissues and may lead to cutting the nerve fascicles. Rarely, the ends of the severed nerve cannot be re-established and abnormal proliferation of Schwann cells results in the formation of a mass of neural elements, named traumatic neuroma [5-6]. The symptoms may vary. This may appear as a painful or painless swelling; however sensory anomalies such as paresthesia may be noticed [7]. Although mucocele is a common lesion in the oral cavity and its treatment is surgery, only one case of mucocele accompanied by traumatic neuroma has been reported. The first case was a 15-year-old girl who complained of painless recurrent swelling in her lower lip, 10 months after treatment of a mucocele with laser surgery and cryosurgery [5]. Our patient was the second and similar to the previous case. Because of the past history of mucocele and its high recurrence rate, the clinical impression of “recurrent mucocele” was mentioned; however, presence of paresthesia was not acceptable. Lip paresthesia is not a common symptom in oral lesions and is normally related to the infections, malignant tumors or surgical treatments [7- 8]. Since the patient did not represent any paresthesia before the first surgery, a strong possibility is that the nerve fiber was cut during the surgery and forming a neuroma was the consequence of this treatment. Surprisingly, despite the large number of surgical treatments which are performed in the oral cavity, few traumatic neuromas have been reported yet. One reason for this might be that severe tissue damage like a nerve cut is required for a neuroma formation [9]. In conclusion, regarding the mucocele as a common mucosal lesion, it is indispensable to treat these lesions conservatively to decrease the post- surgical complications.
